# Adaptive laboratory evolution of *Saccharomyces cerevisiae* enhances butyric acid tolerance and enables co-cultivation with *Clostridium tyrobutyricum*

**DOI:** 10.1186/s12934-026-03078-8

**Published:** 2026-08-03

**Authors:** K. Oehlenschläger, S. Bauschatz, E. Schepp, D. Holtmann, R. Ulber

**Affiliations:** 1 Institute of Bioprocess Engineering, RPTU University Kaiserslautern-Landau, Gottlieb-Daimler-Straße, 67663 Kaiserslautern, Germany; 2 Institute of Process Engineering in Life Sciences, Karlsruhe Institute of Technology, Fritz-Haber-Weg, 76131 Karlsruhe, Germany

**Keywords:** ALE, Acid tolerance, Whole-genome sequencing, Co-culture, *Saccharomyces cerevisiae*, *Clostridium tyrobutyricum*, Process intensification

## Abstract

Adaptive laboratory evolution (ALE) was successfully applied to improve the tolerance of *Saccharomyces cerevisiae* toward butyric acid, enabling its use in co-culture with *Clostridium tyrobutyricum* for the simultaneous production of ethanol and butyric acid as ester precursors. *S. cerevisiae* was adapted through serial transfer at progressively increasing butyric acid concentrations up to 20 g L⁻^1^. The evolved yeast population exhibited significantly enhanced butyric acid tolerance and maintained ethanol production under acid-stress conditions. Interestingly, the evolved population also displayed increased maximum glucose consumption rate and ethanol productivity under non-stress conditions. Whole-genome variant analysis was performed by comparing the wild-type strain, an intermediate evolved population and the final evolved population obtained during ALE. The results suggest that adaptation may be associated with changes in regulatory processes, cellular homeostasis, and membrane and cell wall remodeling. The co-culture with the acid producer *C. tyrobutyricum* demonstrated efficient and balanced substrate utilization, indicating a stable division of labor between the two organisms. Fed-batch cultivation yielded 20.31 ± 2.43 g L⁻^1^ butyric acid and 28.18 ± 3.37 g L⁻^1^ ethanol. With the evolved yeast, ethanol concentrations increased fourfold compared to those achieved with the wild-type strain in previous studies, underscoring the potential of ALE to overcome limitations in co-culture systems.

**Graphical Abstract:**

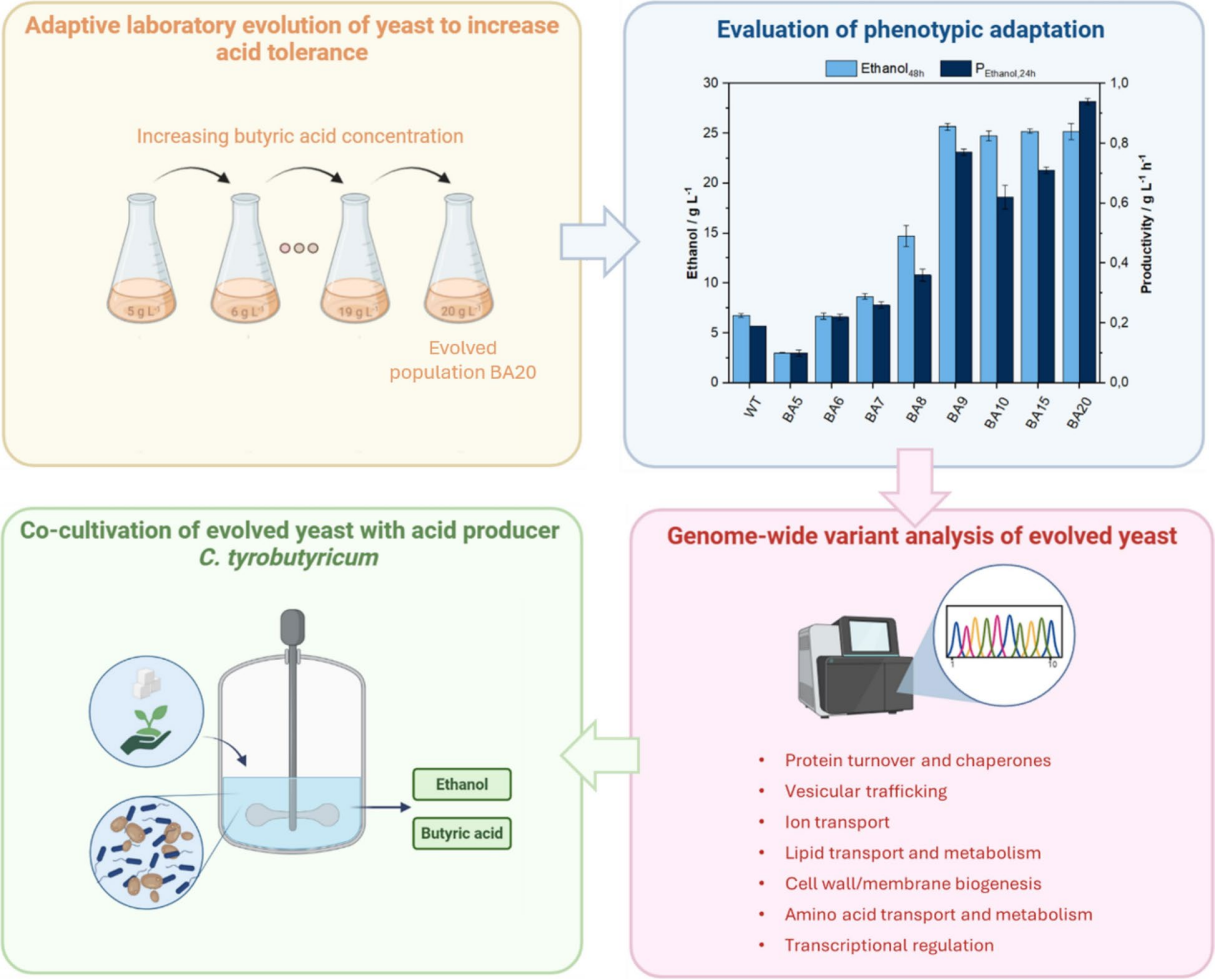

**Supplementary Information:**

The online version contains supplementary material available at 10.1186/s12934-026-03078-8.

## Background

The development of efficient biotechnological processes plays a key role in reducing dependence on fossil resources by enabling the conversion of renewable resources into valuable products. In this context, bioprocesses are under considerable pressure, as they must compete with well-established and highly optimized chemical production routes. To advance bioprocesses, the concept of process intensification promotes the implementation of innovative and highly integrated process concepts [1]. In a previous study, an integrated process for ester synthesis was demonstrated, combining a co-culture system of *Clostridium tyrobutyricum* and *Saccharomyces cerevisiae* with an enzymatic esterification step and in-situ product recovery via liquid–liquid extraction [15]. This process successfully combines microbial production of ethanol and butyric acid with enzymatic esterification, resulting in high concentrations of ethyl butyrate of up to 24 g L¯^1^ extracted into the organic phase. However, the limited acid tolerance of the yeast proved to be a bottleneck in this system, necessitating an initial yeast cultivation phase prior to inoculation of the acid-producing *C. tyrobutyricum*, which extended the overall process duration. In particular, the accumulation of organic acids leads to intracellular acidification and metabolic stress in yeast [2]. To address this limitation, adaptive laboratory evolution (ALE) has emerged as a promising strategy to improve *S. cerevisiae* acid tolerance, as demonstrated in several successful studies (Table 1).

**Table 1:** Overview of adaptive laboratory evolution (ALE) strategies for improving acid tolerance in *S. cerevisiae*

Selective pressure	ALE strategy	Observed mechanism	References
Succinic acid	Serial transfer under gradually increasing acid stress with transfer intervals adjusted to growth; adaptation from 30 to 45 g L¯^1^ succinic acid	Changes in cell wall; thicker and remodeled cell wall with altered mannose and β-1,3-glucan chains	Qin et al. [17]
Acetic acid	Serial transfer with alternating cycles in acid-containing and acid-free medium under gradually increasing acid stress; evolved for ~ 200 generations; adaptation from 3.6 to 7.2 g L¯^1^ acetic acid at pH 4	Adaptation of transport processes, ion homeostasis and stress-related transcriptional regulation; improved control of intracellular pH, more efficient export of acids	Sánchez-Adriá et al. [20]
Acetic acid and low pH	Serial transfer under gradually increasing acetic acid and pH stress with transfer intervals adjusted to growth; 816 generations; adaptation from 3 to 6 g L¯^1^ acetic acid at pH 3, and further to 12 g L¯^1^ at pH 4	Reprogramming of global stress regulation; improved intracellular pH control, energy economy and oxidative stress resistance	Salas-Navarrete et al. [19]
Dicarboxylic acids (glutaric acid, adipic acid, pimelic acid)	Serial transfer under gradually increasing acid stress with transfer intervals adjusted to growth; adaptation from 8 to 30 g L¯^1^ glutaric acid, 4 to 16 g L¯^1^ adipic acid, and 6 to 16 g L¯^1^ pimelic acid at pH 3.2	Upregulation of specific multidrug resistance transporters; enhanced export of acid anions from the cytosol	Pereira et al. [16]

Serial transfer of the cells under progressively increasing acid stress allowed the accumulation of genetic adaptations, resulting in enhanced tolerance to various acids, including dicarboxylic acids (glutaric, adipic, and pimelic acid), succinic acid, and acetic acid, as well as to low pH conditions. The genetic changes were linked to functional adaptations that reprogrammed global stress responses, modified membrane and cell wall characteristics, and adjusted transport processes, ultimately improving intracellular pH homeostasis, enhancing acid export, and increasing stress resistance. Accordingly, the adapted yeast demonstrates increased acid resistance through reduced cell envelope permeability and a more efficient stress response. These fundamental mechanisms underlying acid tolerance were also identified in chemogenomic studies of acid tolerant yeast strains [12, 14]. Compared to rational engineering approaches, ALE enables the selection of complex, multi-genic adaptations that are difficult to predict [4]. This allows the targeted adaptation of a strain to specific process conditions.

In this study, ALE was applied to enhance the butyric acid tolerance of *S. cerevisiae*. Phenotypic adaptation of evolved yeast cells was comprehensively investigated over the course of ALE. Genomic sequencing was employed to investigate genetic changes associated with increased acid tolerance. Finally, the evolved yeast cells were employed in co-cultivation with *C. tyrobutyricum* for the combined production of ethanol and butyric acid. Thus, this work extends the application of ALE beyond improving acid tolerance alone by demonstrating its direct relevance for stabilizing and intensifying a productive yeast–bacterial co-cultivation process. By linking evolutionary strain engineering with process integration, this work aims to contribute to the development of more robust and intensified biotechnological production systems.

## Materials and methods

### Microorganisms

*Clostridium tyrobutyricum* DSM 2637 and *Saccharomyces cerevisiae* DSM 3799 were obtained from DSMZ (German Collection of Microorganisms and Cell Cultures GmbH, Braunschweig, Germany). *Clostridium* cells were stored in a 40% aqueous glycerol solution at -80 °C under anaerobic conditions, while yeast cells were preserved aerobically under the same conditions. The wild-type strain of *S. cerevisiae* was used as the parental strain for adaptive laboratory evolution experiments.

### Media and cultivation conditions

Pre-cultures of *S. cerevisiae* were inoculated with 1% v/v cryoculture. The yeast pre-cultures were incubated under aerobic conditions in 300 mL shake flasks with baffles, each containing 100 mL of pre-culture medium, for 17 h at 32 °C and 150 rpm in a shake incubator (Infors HAT, Ecotron, Infors AG, Bottmingen, Switzerland). The YPG medium was used as the pre-culture medium, which was composed as follows per litre: 10 g yeast extract, 20 g peptone and 20 g glucose. Yeast acid tolerance was assessed based on growth, glucose consumption, and ethanol production at different butyric acid concentrations. Therefore, main cultures were inoculated with 10% (v/v) of yeast pre-culture. Cultivation took place at 37 °C and 150 rpm in 150 mL serum bottles (working volume of 100 mL) closed with butyl rubber stoppers and aluminium crimps in an incubator. A temperature of 37 °C was chosen to match the conditions used for co-cultivation. However, it has been shown that the temperature difference between 32 and 37 °C has no significant effect on ethanol production by *S. cerevisiae* [15]. YPG medium was used as the main culture medium or YPG medium supplemented with different concentrations of butyric acid. In all yeast cultivations, the initial glucose concentration was 50 g L¯^1^, and the initial pH was set to 6.0, with no pH control during cultivation. Maximum productivity P_max_ and glucose consumption rate r_s,max_ were calculated as the maximum slope between consecutive data points. Specific rates (q_s_ and q_P_) were determined based on the average biomass within the considered time interval. For biomass calculation cell dry weight (CDW) was estimated based on an experimentally determined linear correlation between optical density (OD) and CDW (CDW = 0.35∙OD + 0.05, R^2^ = 0.99).

The cultivation of *C. tyrobutyricum* in the pre-culture was carried out under anaerobic conditions in 150 mL serum bottles, each containing 100 mL of modified PY + X medium. The modified PY + X medium consisted of the following components per litre: 10 g yeast extract, 5 g meat peptone, 5 g tryptone, 5 g glucose and 40 mL salt solution. The salt solution contained 0.25 g CaCl_2_ × 2H_2_O, 0.50 g MgSO_4_ × 7H_2_O, 1 g KH_2_PO_4_, 1 g K_2_HPO_4_, 10 g NaHCO_3_ and 2 g NaCl per litre. The pH value was corrected by adding 1 M HCl solution to achieve a pH value of 5.5. To ensure anaerobic conditions, the medium was flushed with nitrogen for 30 min before cultivation began. The pre-culture was then inoculated with 1% v/v of the cryoculture and incubated for 24 h at 150 rpm and 37 °C in a shaking incubator.

### Adaptive laboratory evolution strategy for yeast

**Fig. 1:**
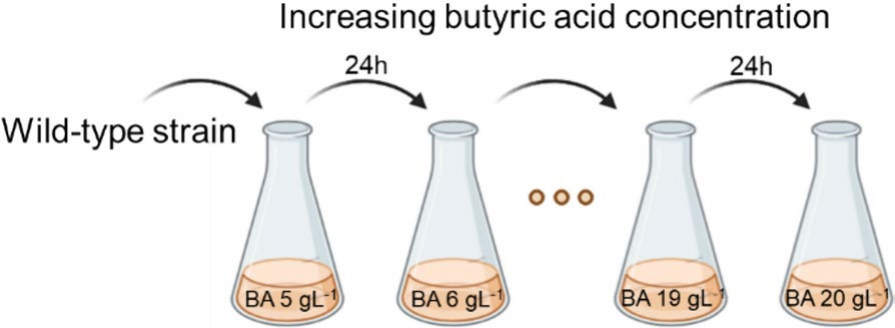
Schematic representation of the adaptive laboratory evolution (ALE) strategy used to improve butyric acid tolerance of *S. cerevisiae;* BA: butyric acid

The WT *S. cerevisiae* strain (DSM 3799) served as the parental strain for yeast cell adaptation using ALE. ALE was performed through repeated serial transfers with gradually increasing acid concentration (Fig. 1).

First, a pre-culture was grown with 1% v/v of the WT yeast strain in a 300 mL shake flask with 100 mL YPG pre-culture medium at 32 °C and 150 rpm. The subsequent adaptive laboratory evolution of the yeast cells was also carried out under aerobic conditions in shake flasks to maximize cell growth and thereby increase the number of generations accumulated during ALE. The YPG medium served as the base medium, to which defined concentrations of butyric acid were added (pH_0_ = 6.0) depending on the respective selection stage. Starting at 5 g L¯^1^ butyric acid, the concentration was increased by 1 g L¯^1^ per selection stage, resulting in a total of 16 selection stages. After 24 h of incubation, part of the fermentation broth was centrifuged for 2 min at 4500 rpm (Centrifuge Z383K, Hermle Labortechnik GmbH, Wehingen, Germany). The pellet was washed two times with 0.9% NaCl solution and was then resuspended in fresh YPG medium with a butyric acid concentration 1 g L^−1^ higher. The culture volume was adjusted prior to centrifugation to achieve an initial optical density of approximately 1 at the beginning of each new stress stage, ensuring comparable starting conditions. At each selection stage, part of the population was preserved as cell stocks, yielding the adapted yeast populations BA5–BA20. Cell stocks were prepared by mixing 0.5 mL of cell suspension with 0.5 mL of 80% (v/v) glycerol solution in cryotubes and stored at − 80 °C. The number of generations was calculated based on optical density (OD) measurements according to Eq. (1).

1 $$n=\frac{\mathrm{ln}\left(\frac{{OD}_{final}}{{OD}_{start}}\right)}{\mathrm{ln}2}$$

### Genome sequencing

Genomic DNA of the wild-type yeast strain, population BA9 and population BA20 was analyzed using Illumina whole-genome sequencing. Therefore, cryopreserved cells from the ALE experiment were incubated in YPG medium (20 g L glucose) in 300 mL shake flasks with baffles, each containing 100 mL medium, at 32 °C and 150 rpm until reaching the exponential growth phase (17 h), then centrifuged and washed twice with 0.9% NaCl. The resulting cell pellet was stored in Eppendorf tubes at − 80 °C and subsequently shipped on dry ice. Library preparation, sequencing, read mapping, and bioinformatic analyses were carried out by Novogene Co., Ltd (Martinsried, Germany). Detailed technical information provided by Novogene has been included in the supplementary material. Sequencing reads from BA9 and BA20 were mapped to the de novo assembled genome of the parental wild-type strain generated in this study, which was used as the reference for variant calling. To identify genetic changes associated with adaptation to butyric acid stress, single nucleotide polymorphism (SNPs) and structural variants were filtered based on their frequency dynamics across populations. Since BA9 and BA20 were sequenced as evolved populations rather than clonal isolates, genotype calls were interpreted at the population level rather than as representing individual clones. Particular attention was given to mutations absent in the wild-type, occurring at intermediate frequencies in BA9, and reaching fixation or near fixation in BA20, suggesting progressive enrichment during adaptive evolution. Further analysis of data was performed using g:Profiler [9].

### Co-cultivation

The co-cultivation of *S. cerevisiae* and *C. tyrobutyricum* was carried out under pH-controlled conditions at pH 6.0, 37 °C and 200 rpm in a 1 L scale bioreactor (HABITAT Research Bioreactor Systems, IKA Werke GmbH & Co. KG, Staufen im Breisgau, Germany). The MP2opt medium described by Engel et al. was used, which is an optimized synthetic clostridial medium based on the P2 medium [5]. The medium consisted of 1.9 g (NH₄)₂SO₄, 0.5 g KH_2_PO_4_, 0.5 g K_2_HPO_4_, 0.2 g MgSO_4_∙7 H_2_O, 0.015 g Fe(II)SO_4_∙7H_2_O, 0.01 g MnSO_4_xH_2_O and 1 mL vitamin solution per litre [5]. The vitamin solution consisted of 2 mg 4-aminobenzoic acid, 2 mg thiamine HCl and 0.01 mg D-biotin per litre. Co-cultivation was carried out as a fed-batch with an initial glucose concentration of 50 g L¯^1^ glucose, whereby the glucose concentration was maintained between approximately 20 and 40 g L¯^1^ through continuous substrate addition over the entire fermentation period. At the start of fermentation, 10% v/v of the yeast pre-culture was added to the medium. After 20 min, 10% v/v of the *Clostridium* pre-culture was added to the system. OD was measured to monitor the overall biomass during co-cultivation and therefore represents the combined growth of both *S. cerevisiae* and *C. tyrobutyricum*.

### Analytical methods

Samples were sterile-filtered and analysed by high-performance liquid chromatography (HPLC) using an Aminex HPX-87H column (300 × 7.8 mm, Bio-Rad Laboratories GmbH, Feldkirchen, Germany) coupled with a refractive index detector (PN3140, Postnova, Landsberg am Lech, Germany). The mobile phase consisted of 2.5 mM H_2_SO_4_ at a flow rate of 0.6 mL min⁻^1^. Yields were calculated based on consumed glucose, with co-culture yields determined from the sum of ethanol and butyric acid concentrations. Standard deviations were calculated from three biological replicates. Statistical analyses were carried out using MATLAB (MathWorks, Natick, MA, USA) software. Statistical significance between the evolved yeast and the wild-type strain was assessed using Welch's two-sample t-test (two-sided) based on three independent biological replicates. Differences were considered statistically significant at *p* < 0.05.

## Results

### Development of an acid-tolerant yeast

**Fig. 2:**
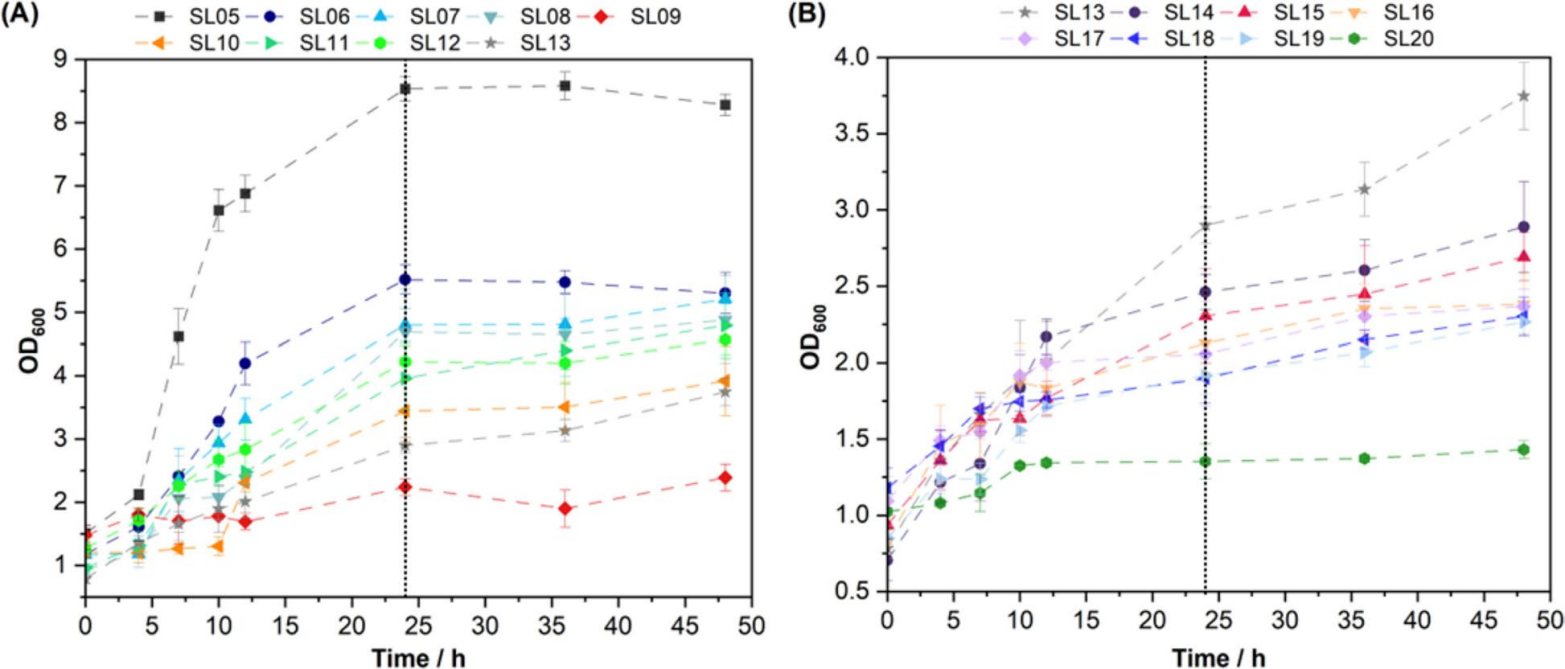
Growth of *S. cerevisiae* during ALE at different selection levels (SL) with increasing butyric acid concentration. SL number indicates the butyric acid concentration in g L¯^1^. **A** SL05–SL13; **B** SL13-SL20. The dotted line indicates the time point of transfer to a new SL. YPG medium supplemented with butyric acid; V = 100 mL; T = 32°C; 150 rpm; pH₀ = 6.0; aerobic conditions; n = 3

Previous studies have shown that the performance of *S. cerevisiae* in a co-culture with *C. tyrobutyricum* is limited by its low tolerance to butyric acid [15]. Inhibitory effects were observed at concentrations as low as 5 g L¯^1^ with ethanol production being almost completely suppressed at 15 g L¯^1^ (see also Fig. 5). To address this limitation, *S. cerevisiae* was subjected to ALE under defined selective pressure to enhance its tolerance to butyric acid. Considering the screening of acid stress tolerance, the ALE was initiated with a selection pressure of 5 g L¯^1^ butyric acid. The evolution was continued for 15 serial transfers with incremental increases of 1 g L¯^1^, reaching a final acid concentration of 20 g L¯^1^ (selection level SL5-20). Yeast cells were transferred to the next selection level after 24 h of cultivation. The evolved yeast populations were designated BA5-BA20 according to the butyric acid concentration applied during their evolution. Growth in the individual selection levels was monitored based on optical density (OD) and is shown in Fig. 2.

A progressive decrease in growth was observed with increasing selection pressure during ALE, with selection level 9 representing an exception, as a significant decline in growth was observed at this level. Based on the OD measurements, a total of around 25 generations evolved over the course of the ALE, reflecting limited growth under the applied stress conditions (Eq. 1, Fig. 2). Under such severe stress conditions, a substantial fraction of the population is unlikely to survive, leaving only a subset of viable cells that contribute to growth. In this case, evolutionary adaptation may be more strongly influenced by cell survival than by cell replication.

**Fig. 3:**
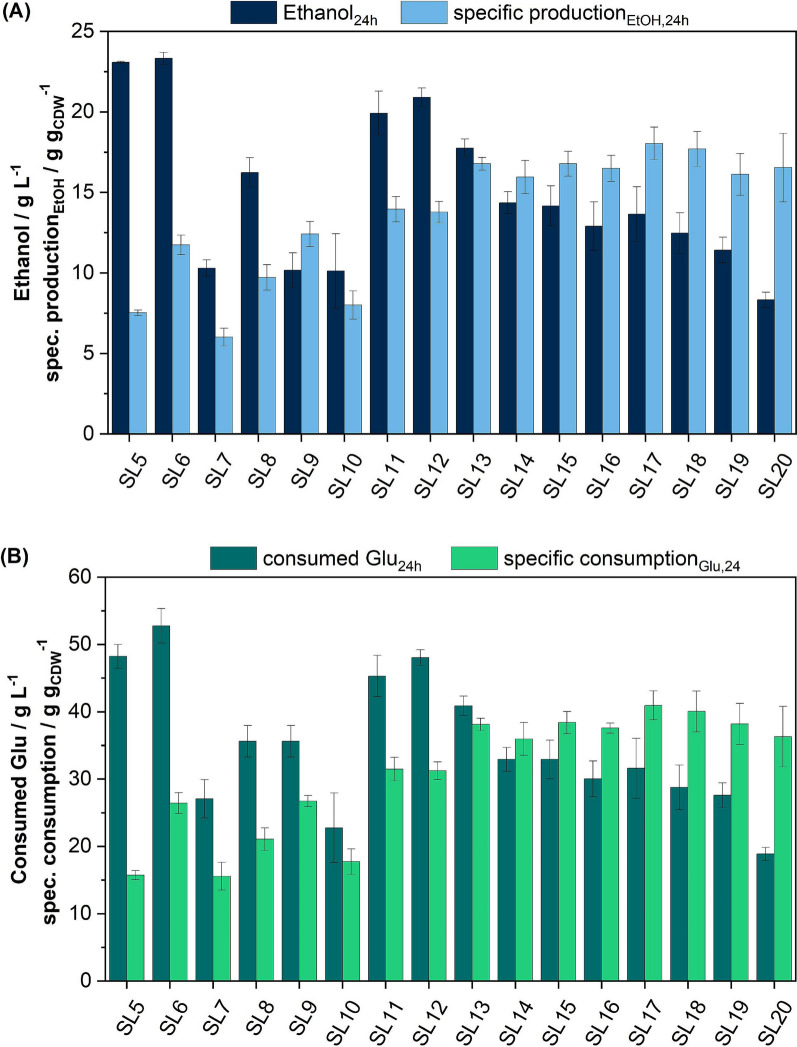
Substrate consumption and product formation during ALE at different selection levels (SL) with increasing butyric acid concentrations. **A** Ethanol production; **B** Glucose consumption. SL number indicates the butyric acid concentration in g L¯^1^. Specific values denote product formation and substrate consumption per biomass at 24 h. CDW derived from correlation with OD. YPG medium supplemented with butyric acid; V = 100 mL; T = 32°C; 150 rpm; pH₀ = 6.0; aerobic conditions; n = 3

To gain further insights into the dynamics during ALE, product formation and substrate consumption were also analysed (Fig. 3). No elevated concentrations of by-products such as glycerol (max. 2 g L¯^1^) or acetic acid (max. 0.5 g L¯^1^) were detected at any ALE level.

A clear decrease in ethanol production and substrate uptake is observed at an acid concentration of 7 g L¯^1^ as a response to elevated stress conditions. The pronounced fluctuations observed during the early stages of ALE may reflect population heterogeneity or transient physiological instability under increasing acid stress. In the subsequent selection levels 11–20, both the specific ethanol production and the glucose consumption stabilize at a constant level, while absolute ethanol production and substrate consumption decline. This observation may indicate the progressive selection of a stress-tolerant subpopulation capable of maintaining metabolic activity at high acid concentrations. However, elevated acid concentrations limit biomass formation, leading to decreased absolute ethanol production and substrate consumption (see Fig. 2).

**Fig. 4:**
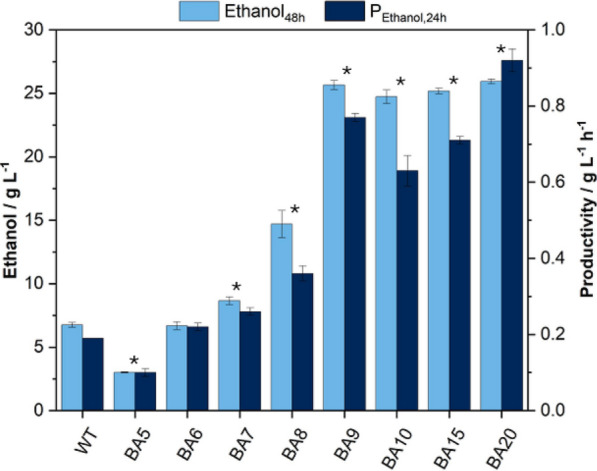
Evaluation of acid tolerance at different stages of ALE; P_24h_ represents the average volumetric ethanol productivity calculated over a 24 h interval; asterisks indicate significant differences compared to the wild-type strain (p < 0.05);YPG medium supplemented with 10 g L¯^1^ butyric acid; V = 100 mL; T = 37°C; 150 rpm; pH₀ = 6.0; sealed serum bottles; n = 3

Cells from each ALE stage were cryopreserved, revived in a non-selective preculture, and subsequently cultivated in medium supplemented with 10 g L¯^1^ butyric acid to compare their adaptation to butyric acid at a constant stress level (Fig. 4).

**Fig. 5:**
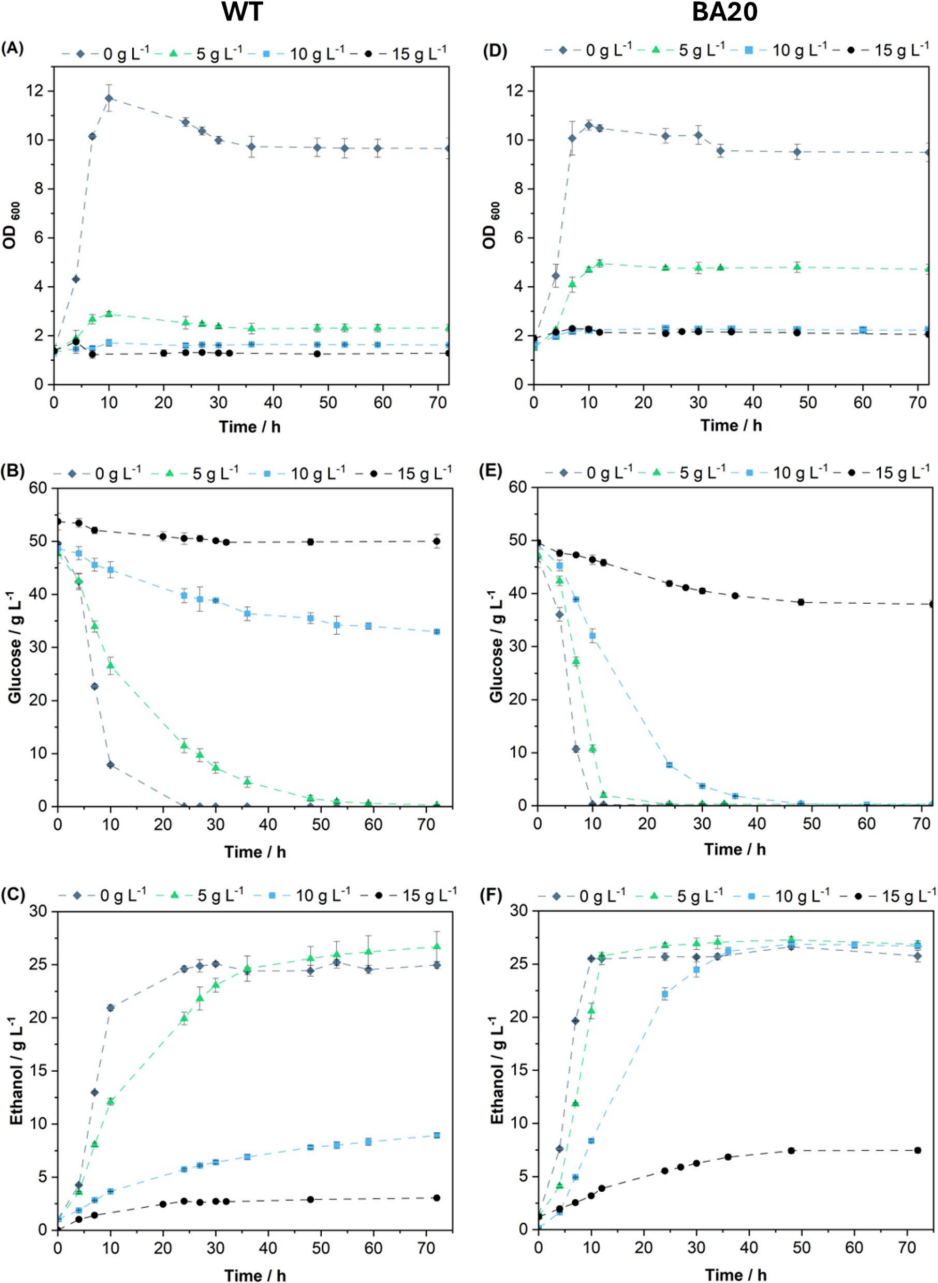
Comparison of cultivation performance under 10 g L¯^1^ butyric acid stress between the wild-type (**A**–**C**) and the evolved BA20 cells (**D**–**F**). V = 100 mL; sealed serum bottles; T = 37°C; 150 rpm; pH₀ = 6.0; n = 3

Ethanol production of the evolved populations is initially lower than that of the wild-type but increases stepwise over the course of the ALE. This initial decrease may be attributed to the fact that the cells originate from stressful conditions and are not yet optimally adapted to the imposed environment, resulting in reduced performance. This is followed by a gradual improvement in tolerance as beneficial mutations accumulate during ALE, ultimately resulting in the selection of a more tolerant yeast population. Notably, ethanol space–time yield shows a pronounced increase resulting from BA9, reaching 0.77 ± 0.01 g L¯^1^ h¯^1^, which is double the productivity observed in the previous ALE level. Considering the observed changes in growth and production during SL9 (see Figs. 2, 3), the intermediate population BA9 may represent a transitional stage during adaptation. Compared to the wild-type, the evolved populations BA7-BA20 show a significantly enhanced ethanol productivity under butyric acid stress, confirming successful adaptation (p < 0.05, Welch’s t-test for independent samples). Cells of the evolved end population BA20 were additionally tested under varying butyric acid concentrations and compared to the wild-type cells. Therefore Fig. 5 shows growth, ethanol production and glucose consumption in direct comparison. The formation of by-products, such as acetic acid and glycerol, is not shown due to their low concentrations (< 0.5 g L¯^1^) for clarity.

Overall, the evolved yeast population BA20 exhibited markedly enhanced productivity under acid stress compared to the wild-type cells (see also Table 2).

**Table 2:** Comparison of maximum substrate consumption rate (r_s,max._) and maximum productivity (P_max._) of WT *S. cerevisiae* and BA20 cells at different butyric acid (BA) concentrations

BA concentration/g L^−1^	WT		BA20	
	r_s,max_/g_Glu_ L¯^1^ h¯^1^	P_max_/g_EtOH_ L¯^1^ h¯^1^	r_s,max_/g_Glu_ L¯^1^ h¯^1^	P_max_/g_EtOH_ L¯^1^ h¯^1^
0	6.56 ± 0.50	2.91 ± 0.04	8.44 ± 0.24	4.02 ± 0.06
5	2.87 ± 0.44	1.49 ± 0.03	5.48 ± 0.09	2.91 ± 0.22
10	0.72 ± 0.44	0.41 ± 0.02	2.28 ± 0.44	1.14 ± 0.04
15	0.29 ± 0.17	0.25 ± 0.01	0.50 ± 0.28	0.35 ± 0.03

Notably, the evolved population showed accelerated ethanol production even without acid stress, resulting in a significantly higher maximum ethanol productivity. Similarly, an increase in the maximum glucose consumption rate was observed in the absence of acid. This suggests that adaptation to acid stress not only improved butyric acid tolerance but also enhanced ethanol production. Beyond that, BA20 sustained ethanol production at 5 g L¯^1^ butyric acid, with no significant difference compared to the stress-free condition. Further increase in acid stress leads to a reduction in ethanol production. However, at 15 g L¯^1^ butyric acid, BA20 produced approximately twice the ethanol concentration of the wild-type strain under the same stress conditions (*c*_EtOH,BA20_ = 6.25 ± 0.25, *c*_EtOH,WT_ = 3.04 ± 0.10). Moreover, it was observed that both, the wild-type strain and the evolved population BA20, produced high ethanol concentrations despite substantial reductions in growth, prompting a comparison of specific consumption and specific productivity (Table 3).

**Table 3:** Comparison of maximum specific substrate consumption rate (q_s,max._) and maximum specific productivity (q_P,max._) of WT *S. cerevisiae* and BA20 cells at different butyric acid (BA) concentrations. CDW derived from correlation with OD

BA concentration/g L^−1^	WT		BA20	
	q_s,max_ / g_Glu_ g_CDW_¯^1^ h¯^1^	q_P,max_ / g_EtOH_ g_CDW_¯^1^ h¯^1^	q_s,max_ / g_Glu_ g_CDW_¯^1^ h¯^1^	q_P,max_ / g_EtOH_ g_CDW_¯^1^ h¯^1^
0	2.25 ± 0.20	1.11 ± 0.01	3.23 ± 0.11	1.53 ± 0.02
5	3.36 ± 0.39	1.76 ± 0.11	4.34 ± 0.19	2.23 ± 0.16
10	1.31 ± 0.76	0.56 ± 0.05	2.74 ± 0.54	1.39 ± 0.08
15	0.54 ± 0.40	0.44 ± 0.09	0.66 ± 0.37	0.42 ± 0.04

Interestingly, maximum specific substrate consumption rate and productivity initially increase with increasing butyric acid concentrations for both yeasts, suggesting an upregulation of metabolic activity to compensate for acid stress. BA20 cells exhibited overall higher productivity and maintained their metabolic activity at increased acid concentrations, indicating enhanced acid resistance and a more effective stress response.

**Fig. 6:**
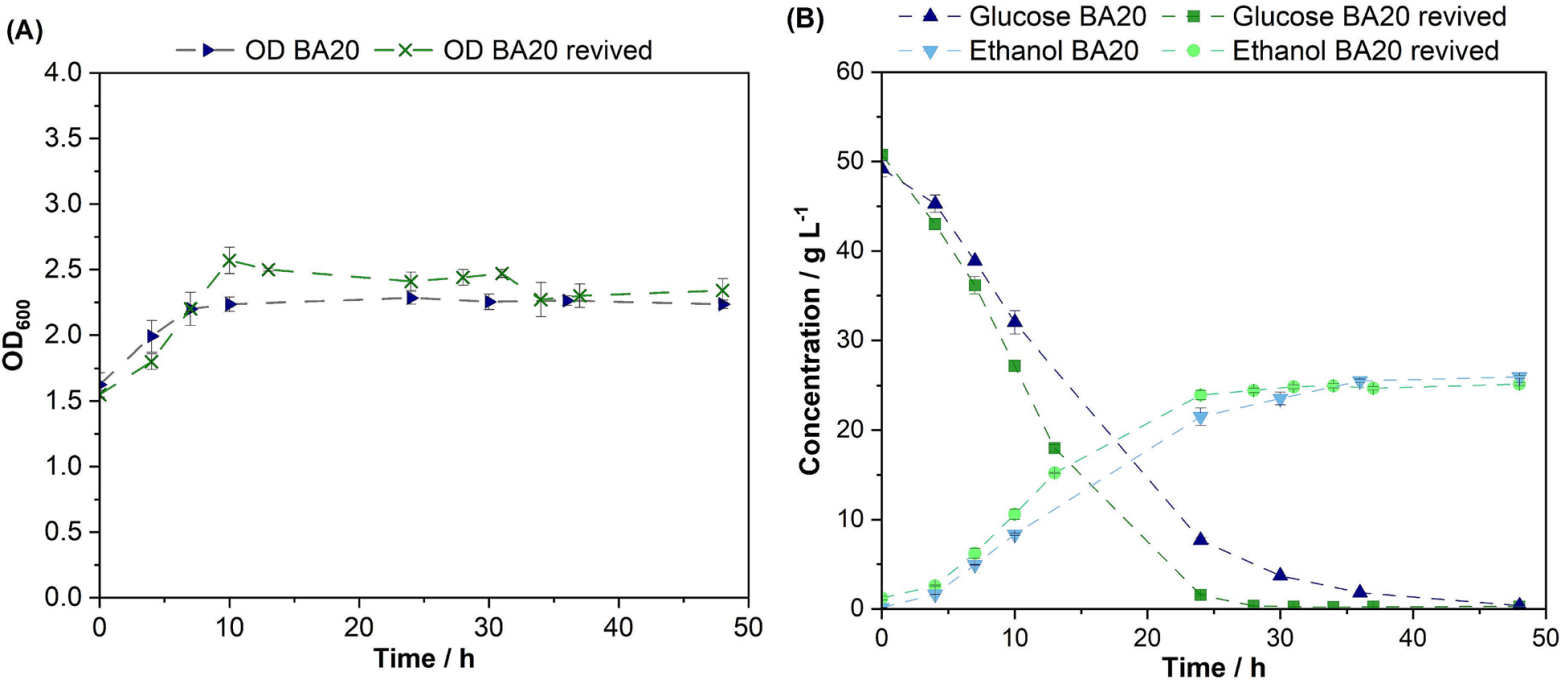
Phenotypic stability of the evolved BA20 cells. **A** Growth curves and **B** Glucose consumption and ethanol production. V = 100 mL; T = 37°C; 150 rpm; pH₀ = 6.0; anaerobic conditions*;* n = 3

To assess phenotypic stability, preserved BA20 yeast cells were used to inoculate a pre-culture (1 vol%) and grown to the exponential phase (17 h). Cells from this cultivation were cryopreserved, subsequently propagated in a pre-culture (inoculum size of 1 vol%) without selective pressure, and finally re-evaluated for their butyric acid tolerance at an acid concentration of 10 g L¯^1^ (Fig. 6).

It is evident that the growth curves, as well as substrate consumption and product formation, show very similar trends. The average productivity achieved after 24 h was 0.95 ± 0.01 g L¯^1^ h¯^1^, which is in good agreement with that of the originally evolved BA20 population (0.92 ± 0.03 g L¯^1^ h¯^1^, Fig. 4). However, additional studies would be required to assess the long-term stability of the evolved population. This confirms that the cells maintained their high acid tolerance under these conditions and demonstrates that the phenotype can be reliably reproduced from preserved stocks. To verify whether the observed phenotypic adaptation also represented a genetic adaptation, genomic sequencing was performed.

### Analysis of genetic changes during adaptive laboratory evolution

Whole-genome sequencing and subsequent variant analysis were performed on the wild-type strain (reference), the intermediate population BA9, and the final evolved population BA20 to capture genetic changes occurring during adaptation. To explore genetic changes potentially associated with adaptation to butyric acid stress, non-synonymous SNPs in coding regions that emerged during adaptive laboratory evolution were analysed. A complete list of the identified variants is available in Table S1 in the supplementary material. Gene Ontology (GO) enrichment analysis was performed using the g:Profiler g:GOSt tool [9]. Strong enrichment was observed for broad GO categories, including binding, catalytic activity, cellular process, and intracellular anatomical structure (adjusted p-values ranging from ~ 10¯^50^ to < 10¯^80^, Supplementary Figure S1). However, driver terms identified by g:Profiler were used to refine more specific functional categories. Among these categories, functions related to membrane transport [e.g., export across plasma membrane (p_adj._ = 1.26∙10¯^3^) and metal ion transport (p_adj._ = 4.98∙10¯^2^], membrane trafficking [clathrin-dependent endocytosis (p_adj._ = 4.69∙10¯^2^)], and cell wall-associated glycosylation [UDP-glucosyltransferase activity (p_adj._ = 9.19∙10⁻^3^)] appear particularly relevant in the context of butyric acid tolerance.

To gain further insights, structural variants were analysed to identify high-impact mutations. A total of 47 genes were identified, of which Table 4 highlights those with a direct association to acid tolerance (full list in Supplementary Table S2).

**Table 4:** Structural variants in coding regions associated with adaptation to acid stress. Functional descriptions of genes were obtained from the *Saccharomyces* Genome Database [6]

Functional category	Gene	Function	Subcategory	Type mutation
Protein turnover and chaperones	*JJJ2*	Hsp40 co-chaperone, facilitates Hsp70-mediated protein folding	Chaperone (Folding)	Nonframeshift deletion
	*FPR4*	FKBP-type peptidyl-prolyl isomerase, catalyzes protein folding	Folding enzyme	2 × frameshift insertion
	*PDI1*	Protein disulfide isomerase, forms disulfide bonds in the ER	ER folding	Nonframeshift deletion
	*HSC82*	Hsp90 family chaperone	Chaperone (Folding)	Nonframeshift insertion
	*SSC1*	Hsp70 family chaperone	Chaperone (Folding)	Nonframeshift insertion
	*SLX8*	E3 ubiquitin ligase, mediates ubiquitination of proteins for proteasomal degradation	Ubiquitin system	Frameshift insertion, frameshift deletion
	*UBP7*	Ubiquitin C-terminal hydrolase	Ubiquitin system	Nonframeshift insertion
	*UBP1*	Ubiquitin-specific protease	Ubiquitin system	Nonframeshift deletion
Vesicular trafficking	*USO1*	ER–Golgi vesicle tethering protein, mediates vesicle docking and fusion between the ER and Golgi	Vesicular trafficking	Nonframeshift insertion
	*VPS36*	Component of the ESCRT-II complex, involved in vacuolar sorting	Vesicular trafficking / degradation	2 × frameshift deletion
Ion transport	*FRE3*	Ferric reductase that reduces Fe^3^⁺ to Fe^2^⁺, involved in redox processes	Redox / Ion metabolism	Frameshift insertion
	*HMX1*	Heme oxygenase, involved in iron homeostasis through heme degradation	Iron metabolism	Nonframeshift insertion
	*TRK1*	High-affinity potassium transporter involved in K⁺ uptake and ion homeostasis	Ion transport	Nonframeshift deletion
Lipid transport and metabolism	*MLG1*	Lysophosphatidic acid acyltransferase, catalyzes phospholipid synthesis	Lipid synthesis	Frameshift deletion
	*PLB3*	Lysophospholipase, catalyzes phospholipid degradation	Lipid remodeling	Nonframeshift deletion
Cell wall/membrane biogenesis	*CTS1*	Chitinase, hydrolyzes chitin during cell wall remodeling	Cell wall remodeling	2 × nonframeshift deletion
	*CHS5*	Chitin Synthase-related, involved in the export of specific proteins, such as chitin synthase Chs3p, from the Golgi to the plasma membrane	Vesicular trafficking / cell wall assembly	Nonframeshift deletion
RNA processing	*CDC39*	ATPase activator subunit of the CCR4-NOT core complex involved in transcriptional regulation and mRNA turnover	Transcriptional regulation	Nonframeshift insertion, Nonframeshift deletion
	*POP2*	Subunit of the CCR4-NOT complex involved in mRNA deadenylation and transcription regulation from RNA polymerase II promoters	Post-transcriptional regulation and mRNA degradation	Nonframeshift deletion
	*SEN1*	ATP-dependent DNA/RNA helicase involved in RNA polymerase II transcription termination and RNA processing	Transcription-coupled RNA processing and DNA repair	Nonframeshift insertion
Amino acid transport and metabolism	*GDH2*	NAD⁺-dependent glutamate dehydrogenase, involved in glutamate catabolism and nitrogen metabolism	Glutamate metabolism	Frameshift deletion

In particular, alterations in components of the protein quality control network, including chaperones, folding enzymes, and the ubiquitin–proteasome system, suggest adaptation associated with the maintenance of protein homeostasis. Also, several genes of the Hsp family were identified as targets of structural mutations. Variants associated with vesicular trafficking, ion transport, redox metabolism, lipid metabolism and cell wall structure indicate changes in intracellular transport, membrane remodelling, and ion homeostasis. Furthermore, several genes associated with transcription and mRNA regulation were affected, indicating a possible contribution of transcriptional processes to the adaptation. In addition, several structural variants located in promoter regions were identified and subjected to gene enrichment analysis, followed by driver term analysis to extract the most representative functional categories (Supplementary Table S3, Figure S2). Here, broadly defined functions such as ion binding and catalytic activity were significantly enriched (p_adj._ > 10¯^10^). Consistent with previous findings, driver genes associated with transmembrane transporter activity (p_adj._ = 5.20∙10¯^3^) were enriched, including genes associated with the plasma membrane (e.g., *PMA1*, *PMA2* and *PDR12*), as well as the vacuolar membrane (e.g., *VMA4*, *VMA5*, *VMA6*, *VMA10*, and *VMA16*). Furthermore, ATP hydrolysis activity (p_adj._ = 2.57∙10¯^2^) was identified as a driver term, indicating an impact on active ion transport. Overall, the identified mutations suggest the involvement of processes related to protein homeostasis, membrane and vesicular trafficking, ion transport and homeostasis, lipid and cell wall remodeling, as well as transcriptional and post-transcriptional regulation.

### Integration of the evolved yeast into the co-culture

**Fig. 7:**
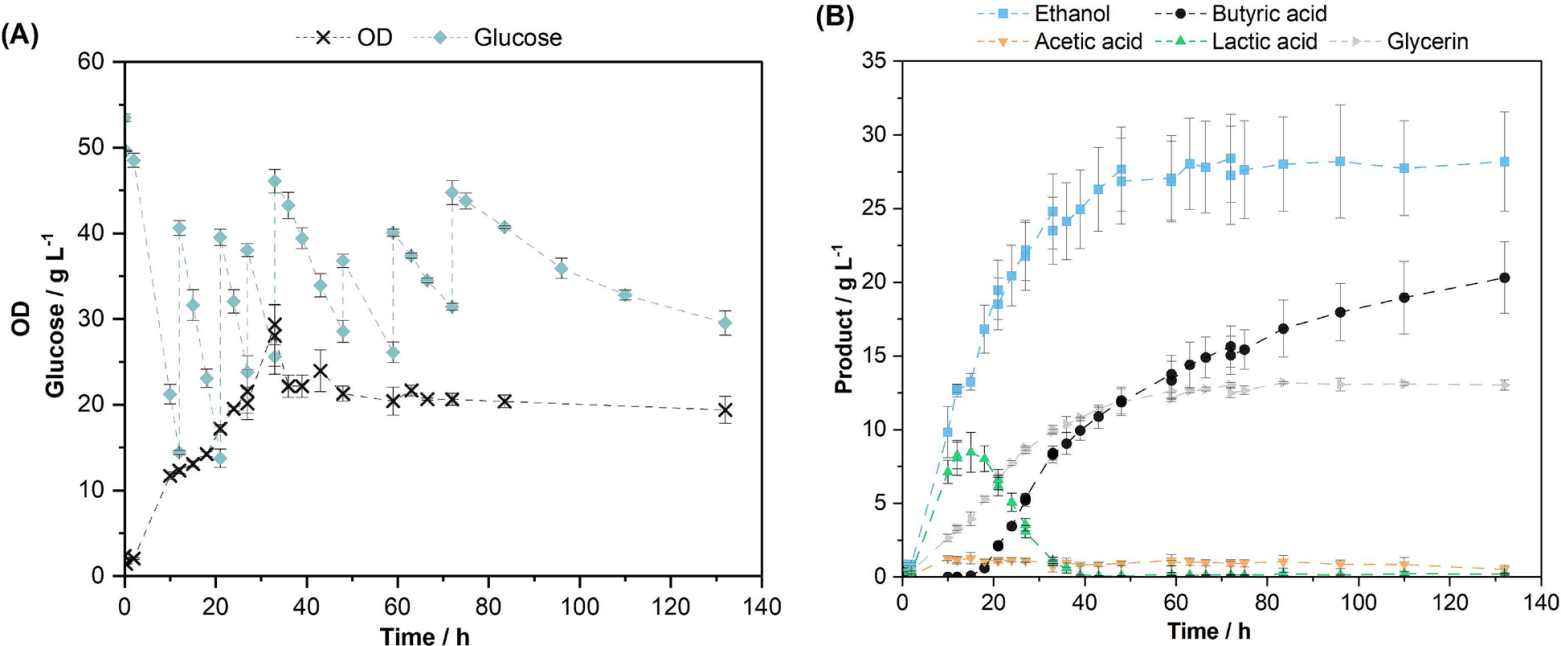
Co-culture of *S. cerevisiae* BA20 und *C. tyrobutyricum*; **A** Optical density and glucose consumption; **B** Product formation; Mp2opt medium; V = 1 L; T = 37°C; 200 rpm; pH controlled to 6 with NaOH; n = 3

The evolved yeast BA20 was employed in co-culture with *C. tyrobutyricum* for the simultaneous production of ethanol and butyric acid. The cultivation conditions were defined based on previous studies and set to 37°C and pH 6 [15]. To achieve high product titers while avoiding substrate inhibition and osmotic stress, the cultivation was performed in fed-batch mode (Fig. 7A). The process was optimized by initial inoculation of the reactors with yeast to establish anaerobic conditions, followed by addition of the strictly anaerobic bacteria after 20 min. The inoculation ratio of yeast to clostridia was set to approximately 2:1 (cell count yeast:clostridia ~ 3.6∙10^6^: ~ 1.8∙10^6^), taking into account the reduced growth of the yeast under anaerobic conditions.

As shown in Fig. 7B, both lactic acid and ethanol production started immediately after inoculation, indicating early metabolic activity of both microorganisms. After approximately 15 h the lactic acid produced by the clostridia was reassimilated and converted into butyric acid. Both main products, ethanol and butyric acid, were then formed simultaneously. Ethanol production was maintained up to a butyric acid concentration of approximately 15 g L⁻^1^, highlighting the increased tolerance of the adapted yeast. Taking into account the dilution caused by glucose and NaOH addition, based on the cumulative recorded feed and NaOH volumes, the produced butyric acid and ethanol were calculated to be 30.42 ± 4.33 g and 40.66 ± 4.11 g, respectively. In comparison, a previous study employing a batch co-cultivation of the wild-type yeast with simultaneous inoculation of *C. tyrobutyricum* resulted in an ethanol concentration of only 10 g L⁻^1^ at a glucose concentration of 100 g L⁻^1^ [15]. This was attributed to the early inhibition of the yeast by butyric acid accumulation. Accordingly, the ALE-derived yeast achieved a fourfold increase in ethanol production. Particularly notable was the formation of the by-products glycerol and lactic acid, which was not observed in the respective monocultures of both microorganisms indicating an altered metabolic behavior in co-culture. Lactic acid, produced by *Clostridium tyrobutyricum*, was detected only at the beginning of the cultivation and was subsequently re-assimilated. In contrast, glycerol, formed by *S. cerevisiae*, accumulated throughout the entire cultivation and reached a final concentration of 13.01 g L⁻^1^. Comparable glycerol concentrations were also observed for the wild-type yeast in co-cultivation and therefore do not represent a trade-off of the ALE adaptation [15]. Overall, these results highlight the effectiveness of the adapted yeast in maintaining metabolic activity under elevated acid concentrations, enabling robust co-culture performance.

## Discussion

In this study the serial transfer of *S. cerevisiae* under progressively increasing butyric acid stress successfully drove adaptive laboratory evolution, resulting a yeast with enhanced acid tolerance. The evolved end population BA20 exhibited significantly increased tolerance to butyric acid and maintained ethanol production at concentrations of up to 10 g L⁻^1^ without pronounced inhibition. A progressive increase in acid tolerance was observed over the course of ALE, consistent with the selection of increasingly tolerant subpopulations. The decline in growth at selection level 9, coinciding with the increase in ethanol productivity under butyric acid stress of BA9 cells, may indicate a strong selection pressure favouring butyric-acid-tolerant phenotypes. The gradual increase in acid tolerance observed at subsequent selection levels is consistent with the progressive selection of tolerant cells within the evolved population. Compared with conventional long-term ALE experiments involving substantially higher numbers of generations (Table 1), the present study was conducted over a comparatively limited number of generations. In contrast to many reported ALE strategies, which adjust transfer intervals to permit recovery and maximize the number of generations achieved under each selection level, our experimental design maintained a continuously high selection pressure. The resulting reduced growth and the associated low number of generations indicate that evolutionary adaptation is driven more by cell survival than by replication, as also described by Voordeckers et al. during adaptation to high ethanol concentrations [22]. Mutagenesis in this context is likely driven predominantly by DNA damage and repair mechanisms rather than replication errors, rendering the number of generations a suboptimal metric for evaluating evolutionary progress. Nevertheless, the pronounced phenotypic improvements observed throughout the experiment indicate that the sustained and progressively increasing selection pressure was sufficient to drive adaptive responses despite the comparatively limited number of generations.

In addition to evolutionary adaptation, the data provide insights into the mechanisms underlying the acid stress response. Increased acid concentrations were associated with elevated substrate uptake and ethanol production in both the wild-type and evolved cells, suggesting an increased energy demand to maintain cellular functions. This mechanism is part of a well-established response to acid stress, whereby energy-generating pathways, particularly glycolysis, are upregulated to support energy-intensive defense mechanisms such as ATP dependent proton pumping [12]. Under anaerobic conditions, the elevated glycolytic flux is linked to increased ethanol production. Interestingly, this increased metabolic activity in BA20 cells was also observed in the absence of acid stress and resulted in accelerated ethanol production compared to the wild-type. Consequently, the evolved yeast is not only more acid-tolerant but also exhibits enhanced ethanol productivity.

Whole-genome sequencing identified candidate genetic changes associated with the observed adaptation of the evolved population. Many of the genes identified in the variant analysis are known to be involved in the cellular response to acid stress. Weak acids such as butyric acid can diffuse across the cell membrane in their undissociated form, leading to cytosolic acidification and necessitating active proton extrusion and ion balancing mechanisms. To counteract intracellular acidification, the plasma membrane H⁺-ATPase actively exports protons from the cytosol, while the vacuolar H⁺-ATPase contributes by sequestering excess protons into the vacuole, thereby supporting the restoration of intracellular pH homeostasis [2, 13]. Here, associated genes such as *PMA1*, *PMA2* were found as structural variants located in promoter regions. In this context, potassium influx provides the necessary counterion flux to sustain proton extrusion and maintain electrochemical balance across the plasma membrane [12, 23]. Therefore, genes involved in ion transport, such as the potassium transporter *TRK1*, contribute to intracellular pH homeostasis [8, 12, 14]. Moreover, mutations were identified in the promoter regions of ABC transporters likely involved in the efflux of weak acid anions [14]. The Hsp family genes identified here are well established as key components of the cellular stress response and play a central role in maintaining protein homeostasis through their function as molecular chaperones [13, 21]. Identification of genes involved in cell wall and membrane biogenesis suggests that structural remodeling may contribute to reduced weak acid permeability. Previous studies on acid tolerance in yeast have shown that lipid remodeling alters membrane composition and properties, thereby modulating membrane integrity and permeability [7]. Similarly, increased acid tolerance has been shown to result from enhanced cell wall stiffness and reduced permeability due to cell wall remodeling, including modifications of β-glucan chains [18]. Together, these results point to candidate processes potentially associated with adaptation involving regulatory network rewiring, ion homeostasis, proteostasis, and cell envelope remodeling and might be similar to that observed for other organic acids, such as acetic acid [12]. This is further supported by a chemogenomic study indicating shared tolerance mechanisms for acetic, butyric, and octanoic acid [14]. However, the identified genetic variants should be regarded as candidate adaptive changes associated with the observed phenotype rather than definitive causal determinants. Further omics-based analyses, together with the isolation and characterization of individual clones from the evolved population, will be required to identify the genetic basis of the observed phenotype and to elucidate the molecular mechanisms underlying enhanced butyric acid tolerance.

In the following step, the evolved yeast BA20 was introduced into co-culture with *C. tyrobutyricum*. In this approach, the facultative anaerobic metabolism of the yeast was leveraged to establish anaerobic conditions prior to the introduction of *Clostridium*. This eliminates the need for prior reactor anaerobization and enhances process robustness against oxygen ingress, for example during nutrient feeding. The observed simultaneous production of ethanol and butyric acid indicates stable coexistence and concurrent metabolic activity of both microorganisms during co-cultivation. Considering ethanol and butyric acid as the main products, the co-culture achieved an overall product yield of 0.43 ± 0.004 g g⁻^1^, which is significantly higher than the yield achieved in co-culture with the wild-type strain (0.35 ± 0.01 g g⁻^1^) [15]. A total glucose consumption of 165.96 ± 1.22 g L was achieved. Based on product formation, glucose utilization was distributed in a balanced manner between the two organisms, with approximately 48% and 37% allocated to ethanol and butyric acid production, respectively. These results suggest that the co-culture efficiently converted a substantial fraction of the consumed glucose into target products, while maintaining a functional division of labor and stable coexistence between the two organisms. The interaction between the two microorganisms can also be observed in the formation of by-products. Substrate competition in co-cultures can redirect metabolic fluxes toward enhanced glycolysis and overflow metabolism, such as glycerol formation in yeast [3, 11]. Similarly, in *C. tyrobutyricum*, transient lactate formation serves as a NADH-dependent redox-balancing mechanism, likely reflecting increased intracellular NADH/NAD⁺ ratios that may arise from elevated glycolytic flux [24]. However, lactic acid was reassimilated and redirected toward butyric acid synthesis, consistent with reports describing reversible lactate metabolism in *Clostridium* species [24]. Comparable competition effects have been reported in a co-culture of *S. cerevisiae* and *Clostridium acetobutylicum*, where increased substrate uptake and altered redox metabolism enhanced solvent production [10]. Despite the formation of by-products, the co-culture provides significant advantages. Integrating both microorganisms in one system rather than operating two separate cultivation processes represents a promising approach to process intensification. The combined process configuration has the potential to reduce capital and operating costs by avoiding duplicated fermentation infrastructure and associated energy demands. Moreover, metabolic cooperation within the co-culture, where yeast consumes oxygen and contributes to the establishment of anaerobic conditions, thereby eliminating the need for external anaerobization of the medium. In addition, the co-culture simplifies the process scheme by enabling the formation of both ester precursors in a single reactor. Esterification could be directly integrated into the same reactor via enzymatic catalysis, as demonstrated in a previous study [15]. In contrast, a corresponding two-reactor process requires an intermediate mixing step, resulting in dilution of the products and consequently lower concentrations. In turn, the co-culture maintains higher product concentrations, which favorably shift the equilibrium of the subsequent esterification reaction. Whereas previous studies relied on delayed inoculation of the *Clostridium* to compensate for the yeast’s limited acid tolerance, the use of the ALE-derived yeast now enables simultaneous inoculation and simultaneous production of ethanol and butyric acid [15]. This co-culture approach demonstrates how the combination of evolutionary engineering and process integration can unlock new opportunities for intensified bioprocess design.

## Conclusion

In this study, ALE was successfully applied to enhance the tolerance of *Saccharomyces cerevisiae* toward butyric acid, enabling its application in co-culture with *Clostridium tyrobutyricum*. The evolved yeast population BA20 exhibited significantly improved acid tolerance and additionally showed increased productivity even in the absence of acid stress. Genome sequencing indicates that adaptation is associated with changes in regulatory networks, cellular homeostasis, proteostasis, and membrane and cell wall remodeling. The integration of the adapted yeast into co-culture enabled the simultaneous production of ethanol and butyric acid within a single reaction system, demonstrating stable coexistence and efficient substrate utilization. Overall, this work demonstrates that ALE is a powerful strategy to overcome physiological limitations in co-culture systems, enabling novel process configurations and contributing to process intensification.

## Supplementary Information

Supplementary material 1.

Supplementary material 2.

Supplementary material 3.

Supplementary material 4.

## Data Availability

All data discussed in this study is included in this article and its additional file. Additional data are publicly available in the DaRUS repository at 10.18419/DARUS-5936.
