# Supplementary material for: Adaptive laboratory evolution of Saccharomyces cerevisiae enhances butyric acid tolerance and enables co-cultivation with Clostridium tyrobutyricum
Source: Microb Cell Fact. 2026 Aug 3;25:166. doi: 10.1186/s12934-026-03078-8 (PMC13436328; doi:10.1186/s12934-026-03078-8)
Supplement: Supplementary file 1 [file 12934_2026_3078_MOESM1_ESM.docx]

**Supplementary Material**

**Whole-genome sequencing**

Library preparation, sequencing, read mapping, and bioinformatic analyses were carried out by Novogene Co., Ltd (Martinsried, Germany). The following information was provided by Novogene. Whole-genome sequencing of the wild-type strain (WT) and the evolved populations BA09 and BA20 was performed using an Illumina paired-end sequencing platform (2 × 150 bp reads). The assembled genome sequence of the WT strain generated in this study was used as the reference genome. Sequencing quality was high for all samples (Q30 > 96%), with average sequencing depths of 113.5× (WT), 176.4× (BA09), and 196.1× (BA20). Reads were aligned using BWA-MEM. Single nucleotide polymorphisms (SNPs) and insertions/deletions (InDels) were identified using GATK HaplotypeCaller (DePristo et al., 2011) following the GATK Best Practices workflow. Variants were functionally annotated using ANNOVAR (Wang et al., 2010). Structural variants were detected using BreakDancer (Chen et al., 2009) and only variants supported by at least two paired-end reads were retained for further analysis.

**References**

Chen, K, et al. BreakDancer: an algorithm for high-resolution mapping of genomic structural variation. Nature Methods. 2009, 6:677-681.

DePristo M A, Banks E, Poplin R, et al. A framework for variation discovery and genotyping using next-generation DNA sequencing data. Nature Genetics. 2011, 43(5):491-498.

Wang K, Li M, Hakonarson H. ANNOVAR: functional annotation of genetic variants from high-throughput sequencing data. Nucleic Acids Research. 2010, 38(16):e164.

**Gene enrichment analysis**

**Figure 1:** Gene enrichment analysis from non-synonymous SNPs in coding regions performed with g:Profiler (Kolberg et al. 2023). Full Gene list in Table S1.

**Figure 2:** Gene enrichment analysis of structural variants (SVs) located in promoter regions performed with g:Profiler (Kolberg et al. 2023). Full Gene list in Table S3.

**References**

Kolberg, Liis; Raudvere, Uku; Kuzmin, Ivan; Adler, Priit; Vilo, Jaak; Peterson, Hedi (2023): g:Profiler-interoperable web service for functional enrichment analysis and gene identifier mapping (2023 update). In: *Nucleic acids research* 51 (W1), W207-W212. DOI: 10.1093/nar/gkad347.
